# Effect of feeding a diet comprised of various corn silages inclusion with peanut vine or wheat straw on performance, digestion, serum parameters and meat nutrients in finishing beef cattle

**DOI:** 10.5713/ab.21.0088

**Published:** 2021-06-23

**Authors:** Hongrui Zhang, Liyang Zhang, Xiao Xue, Xiaoxia Zhang, Hongyi Wang, Tengyun Gao, Clive Phillips

**Affiliations:** 1 Henan International Joint Laboratory of Nutrition Regulation and Ecological Raising of Domestic Animal, College of Animal Science and Technology, Henan Agricultural University, Zhengzhou 450002, China; 2 Henan Forage Feeding Technology Extension Station, Zhengzhou 450008, China; 3 Nanyang Animal Husbandry Technology Extension Station, Nanyang 473068, China; 4 Curtin University Sustainability Policy (CUSP) Institute, Curtin University, Perth 6845, Australia

**Keywords:** Beef Cattle, Beef Nutrients, Corn Silage, Growth Performance, Ruminal Degradability

## Abstract

**Objective:**

The objective of this study was to compare the feeding value, meat nutrients and associative effects of a diet comprised of various corn silages inclusion with peanut vine or wheat straw in finishing beef cattle.

**Methods:**

One hundred and eighty Simmental crossbred beef steers were blocked and assigned to the follow treatments: i) whole plant corn silage-based diet (control, WPCS), ii) mixed forages-based diet (replacing a portion of corn silage with wheat straw, WPCSW), iii) corn stalklage-based diet (CS), and iv) sweet corn stalklage-based diet (SCS). Each group consisted of 5 repeated pens with 9 steers/pen. The diets were formulated to be isonitrogenous and isoenergetic with same forage to concentrate ratio. Experimental diets were fed for 90 d.

**Results:**

The effective ruminal degradability of dry matter and crude protein were highest for WPCS diet (p<0.05), for neutral detergent fiber was highest in SCS diet (p<0.05). The average daily gain was greater for cattle offered the WPCS diet, intermediate with WPCSW and SCS and lowest with CS (p<0.001). The concentration of non-esterified fatty acid in serum was higher for steers fed with CS and SCS diets than those offered WPCS and WPCSW steers (p<0.001). The treatments did not affect the general nutritional contents and amino acids composition of Longissimus dorsi of steers (p>0.05).

**Conclusion:**

The corn silage-based diet exhibited the highest feeding value. The sweet corn stalklage and wheat straw as an alternative to corn silage offered to beef cattle had limited influence on feeding value and meat nutrients. However, the value of a corn stalklage-based diet was relatively poor. To sum up, when the high quality forage resources, such as corn silage, are in short supply, or the growth rate of beef cattle decreases in the later finishing period, the sweet stalklage and wheat straw could be used as a cheaper alternative in feedlot cattle diet without sharp reducing economic benefits.

## INTRODUCTION

Whole plant corn silage is one of the most important feedstuff due to its high dry matter (DM) yield and digestible fiber. It has been popularized in dairy cattle production around the world and beef cattle production in Europe and North America. However, corn silage is underproductive in some regions such as China and not popular in beef cattle raising [1]. Consumer markets for grass-finished beef continue to expand around the world, but given the limited availability of grassland, research is been conducted to evaluate unconventional forage resources as major components in beef cattle production [2]. Corn stalklage is a common alternative for beef cattle feeding in the districts short of forage resources due to its advantages of large-scale production and low-price [3]. Sweet corn is a kind of new corn with rich nutrition, good palate and high economic benefits. It is sweet and is called “fruit corn”. Sweet corn stalklage is rich in water soluble carbohydrate and has a low buffering capacity due to early harvest, providing a promising forage resource with appropriate fermentation characteristics [4]. Crop straws, such as wheat straw and peanut vine, are commonly used in beef cattle diets. In addition to great yield, peanut vine has a high feeding value, characterizing by its high crude protein (CP) concentration, approximately 12%, and it contains more digestible DM, neutral detergent fiber (NDF), acid detergent fiber (ADF) than alfalfa hay [5].

It is generally recognized that there are associative effects among ruminant feed ingredients. Lu [6] pointed out that associative effects in feed refers to the interaction between nutrients, non-nutrients and anti-nutrients in different feed components. A good combination of forages will exhibit a positive associative effect to improve the utilization of feed. Therefore, the combination of high digestibility and good fermentation effects of feed ingredients should be considered when preparing the diets [7]. The dietary inclusion of corn silage conveys good nutritive value. In a study by He et al [3], corn silage could be substituted for corn stalklage in finishing beef cattle diets with no negative effects on their performance, carcass traits or meat quality. The combination of corn silage and wheat straw also exhibited promising impacts on intake and body weight (BW) gain in beef cattle [8]. Mazzenga et al [9] had verified that stepwise inclusion of conventional corn silage to replace wheat straw in a total mixed ration (TMR) had no adverse effects on health, dry matter intake (DMI) and feeding behavior of Simmental young bulls [9].

Despite the popularity of corn silage, it remains uncertain whether it is superior to corn stalklage, sweet corn stalklage, or whether substituting a portion of corn silage with low quality stover as a cheaper alternative for finishing beef cattle raising may alter carcass and meat nutrients. We hypothesized that reasonable use of medium and low quality forages in the diet could greatly exploit the associative effects, and reduce the feed cost without affecting production performance and meat nutrients. Therefore, the present research was conducted to compare the growth performance, ruminal degradability, serum parameters and meat nutrients of finishing beef cattle feeding diet based on various corn silages, including corn silage, corn stalklage, sweet corn stalklage or substituting a portion of corn silage with wheat straw, combined peanut vine as forage components.

## MATERIALS AND METHODS

### Forage used in the experiment

All forages used in this experiment were harvested in the fall of 2016 from a plantation area in Nanyang (central region of China). Whole plant corn was harvested at the two-third milk line stage using a Claas silage harvester (Claas of America LLC, Columbus, IN, USA). The chop length was set to 1.5 cm and the crop was ensiled into an above-ground horizontal silage silo. Simultaneously, ears of sweet corn (fruit corn) at milk stage were harvested for human consumption and sweet corn stalklage was produced with the fresh stalk (sweet corn plant removed ears) subjected to the same processes as for corn silage. Baled corn stalklage was purchased from a local commercial company, with the fresh conventional corn plant removed ears at full ripe stage ensiled at a theoretical cutting length of 1.5 cm. The dry wheat straw and peanut vine were purchased from the surrounding farmers and stored in a forage warehouse to prevent damage. The chemical compositions of forages used for the study were analyzed as later described.

### Animals, experimental design, and dietary treatments

The feeding trial was carried out in the Shiying Farm of Kerqin Cattle Industry Nanyang Co., Ltd. (Nanyang, China) from May 2017 to August 2017. All experimental procedures were approved by the Animal Care and Use Committee of the Henan Agriculture University (Permit No. HNND2017 031018) and followed the practices of Guide for the Care and Use of Agricultural Animals outlined by Federation of Animal Science Societies [10].

One hundred and eighty Simmental crossbred steers (BW = 427.67±29.35 kg, age = 431±28 d) were blocked in to five groups of 36 steers on the basis of initial BW, and then assigned to identical research pens (4 pens/block; 9 steers/pen), where each of the 4 research diets was assigned to 1 pen within each block. Each group consisted of 5 repeated pens with 9 cattle/pen. The diets were formulated to be isonitrogenous and isoenergetic with same forage to concentrate ratio, as follows (Table 2): i) the whole plant corn silage-based diet (WPCS, used as the control diet), ii) the mixed forages-based diet (WPCSW, formulated by replacing a portion of whole plant corn silage with wheat straw), iii) the corn stalklage-based diet (CS), and iv) the sweet corn stalklage-based diet (SCS). Peanut vine was included as the main dry forage in all four diets, of which is a by-product generated in the process of peanut harvest with large yield and high feeding value. The chemical compositions of the silages and crop straws used in present research are shown in Table 1. The animals were given a 14 day-adaptation period to adapt to the new group members and limited requirements, such as the environment of pen, etc. All steers were given free access to water and control diet for clearing the difference of rumen environment. Before the feeding trial, the steers were weighed to determine the initial BW, and then the feeding trial commenced for 90 days. All the steers were reared with *ad libitum* access to TMR and water.

### *In situ* ruminal degradability

The ruminal degradability of the four experiment diets was determined by the *in situ* nylon bag technique according to Mehrez and Ørskov [11]. The *in situ* trial was conducted in the Experimental Practice Farm and Demonstration Center of Henan Agricultural University (Zhengzhou, China). Three cattle fitted with permanent ruminal fistulae were used. The cattle were fed *ad libidum* a 60:40 forage:concentrate diet (DM basis) which consisted of 44% Chinese wildrye, 16% corn silage, 40% concentrate mixture. The composition (DM basis) of the ration was of 14.4% CP, 49.2% NDF and 30.6% ADF. Animals were fed in equal portions at 08:00 and 16:00 h and had free access to water. Four diets samples were milled and placed (approximately 5 g) in 10×20 cm nylon bags (pore size 40 μm). The bags were incubated into the rumen of the 3 cattle (two bags per time period per cattle) for 0, 4, 8, 12, 16, 24, 36, 48, and 72 h. Following removal from the rumen, bags were washed in cold water until the runoff water was clear. Washed bags were dried at 65°C for 48 h in a fan-assisted oven. Residues from each nylon bag was analyzed for DM, CP, NDF, and ADF contents as follows. Ruminal disappearance was calculated from nutrients concentrations in the original samples and the ruminal residues. The degradation parameters using the equation of Orskov and McDonald [12,13]:


$$\text{P}=\text{a}+\text{b}\times (1-{\text{e}}^{-\text{ct}})$$

Where p is ruminal disappearance at time t (%), a is the soluble fraction (%), b is the slowly degradable fraction (%), and c is the rate at which the b fraction is degraded (%/h).

Effective ruminal degradability (ED) was estimated using the equation of Orskov and McDonald [12]:


ED=a+[(b×c)/(c+k)]

Where k is the estimated ruminal flow rate of 5.0%/h.

### Feed intake and growth performance

Daily TMR and orts from each pen were weighed and sampled every day. All samples were immediately frozen at −20°C until they were analyzed. After thawing, the samples were composited for each 15 d of study, and then the DM contents were analyzed. Cattle were weighed in the morning before the diets were offered at start of the trial and each 30 d thereafter. Calculations of the average daily gain (ADG), DMI, feed conversion ratio (F/G) and economical evaluation were carried out based on the above measurements.

### Laboratory analysis for feed and ort samples

Forage ingredients were sampled every 15 d and kept frozen until later analysis. The composites of TMR, orts and individual dietary forage ingredients were dried to a constant weight at 65°C for 48 h to analyze DM content. Samples of TMR and ingredients were then ground through a 1-mm sieve for chemical analysis. The CP concentration was determined using an automatic distillation and titration system (K9860, Haineng Instrument Co., Ltd, Jinan, China) according to the procedure of Krishnamoorthy et al [14]. The contents of NDF and ADF were analyzed by an ANKOM 220 Fiber Analyzer (ANKOM Technol. Corp., Fairport, NY, USA) with sodium sulfide and heat-stable α-amylase [15]. And the procedures of the Association of Official Analytical Chemists [16] were used to measure the contents of ether extract (method 920.39) and ash (method 942.05). In addition, the concentration of lactate in corn silage used in present work was measured by HPLC method according to Wang and Nishino [17], and the concentrations of volatile fatty acids (VFA) were detected using an ion-Chromatograph (ICS-3000, Dionex Corporation, Sunnyvale, CA, USA). The NH_3_-N concentration was analyzed using the phenol hypochlorite colorimetric method developed by Broderick and Kang [18].

### Serum parameters

Two of 9 steers per pen were randomly selected for collecting blood samples (A total of 10 samples/group) before the morning feeding at 0, 45, and 90 d of the trial. Blood samples were collected from the coccygeal vein into Vacutainer tubes (Huawei Medical Appliances Co. Ltd., Yangzhou, China). The serum fraction was separated and transferred into clean tubes. The concentrations of total protein (TP), albumin (ALB), globulin (GLB), urea nitrogen (UN), glucose (GLU), nonesterified fatty acid (NEFA), creatinine (CREA), and the activity of lactate dehydrogenase (LDH) were analyzed using the corresponding test kit according to the manufacturer’s instructions (Nanjing Jiancheng Bioengineering Institute, Nanjing, China).

### Meat nutrients

One of 9 steers per pen was randomly selected for slaughtering trial (A total of 5 steers/group). The steers were transported to a nearby commercial abattoir, where they were held in lairage overnight and slaughtered as a single group the following day. Sample cores (200 g) from the ninth and tenth rib area of the Longissimus dorsi (LD) were collected from each carcass, trimmed of visible fat. These were then immediately frozen and transported in liquid nitrogen, and stored in a −80°C freezer until analysis. All beef samples were ground to pass through the 1-mm screen to analyze the nutrient compositions. The contents of CP, EE, and ash were measured as described above. The contents of amino acids were analyzed using an automated amino acid analyzer after post-column ninhydrin derivatization (Hitachi L-8900, Hitachi High-Technologies Corporation, Tokyo, Japan).

### Statistical procedures

The data were analyzed using SAS/STAT software (version 9.2, SAS Institute Inc., Cary, NC, USA). Before analysis, daily measurements of intake were adjusted to 15 days means. All data were tested for normality using Anderson-Darling test. The data of feed intake, growth performance and serum biochemical parameters collected from the steers with same pen were adjusted to mean values of pen and then analyzed as a randomized complete block design with repeated measures when applicable by a MIXED model procedure. Model was Y_ijk_ = μ+P_i_+D_j_+B_k_+P_i_×D_j_+ɛ_ijk_, where Y_ijk_ represents the dependent variable; μ, the overall mean; P, the fixed effect of period (i = 1 to 3); D, the fixed effect of diet (j = 1 to 4); B, the random effect of block (k = 1 to 5); P_i_×D_j_, the fixed effect of period and diet; and ɛ_ijk_, the residual error. Data collected on the *in situ* ruminal degradability trial and meat nutrients were analyzed using the general linear model procedure. Statistically significant differences among treatments were assessed using Tukey’s adjustment test. Significance was declared when p<0.05, results were presented as means±standard deviation.

## RESULTS

### Chemical composition of experimental silages and crop straws

The chemical compositions of the silages and crop straws are shown in Table 2. The corn silage had satisfactory chemical composition as shown by its relatively low NDF and ADF content and high starch concentration. It also had a good fermentation profile with a low pH and prevalence of lactate, VFA and NH_3_-N. The VFA content of sweet corn stalklage was relatively low while the CP was the highest of the three corn silages. The corn stalklage exhibited relatively high NDF and ADF contents. Peanut vine, as the main dry forage used in this experiment, had relatively high CP content (12.04%) and low fiber fraction, close to corn silage. As an alternative forage, wheat straw exhibited the highest NDF and ADF content.

### *In situ* ruminal degradability

The parameter estimates of ruminal degradability of DM, CP, NDF, and ADF from the four diets are shown in Table 3. The WPCS diet had a higher soluble fraction for DM, CP, and NDF that was higher than those in WPCSW and CS diets (p<0.05), but it was not different compared with SCS diet. The slowly degradable fraction of DM was higher for WPCSW diet than that in WPCS and SCS diet (p = 0.015). There was no difference for slowly degradable fraction of CP among four diets. However, the slowly degradable fractions of NDF for WPCS, WPCSW and SCS diets were higher than that in CS diet (p<0.05). The WPCSW diet showed highest slowly degradable fractions of ADF and was higher than the other three diets (p<0.05). The CS diet exhibited higher undegradable fraction for all nutrients than those in WPCS and SCS diet (p<0.05). Simultaneously, there was no difference between WPCS and SCS diet for nutrients degradability. The ED of DM and CP were highest for the WPCS diet, followed by the SCS, then WPCSW and finally least for the CS diet, and the differences were significant among four diets (p<0.05). The ED of NDF was higher for SCS diet and lowest for CS diet, the differences among the four diets were significant (p<0.05).

### Feed intake and growth performance

The feed intake, growth performance, F/G on d 0, 30, 60, and 90, and economical evaluation of beef cattle offered various corn silage-based diets are presented in Table 4. Initial BW were similar among diet groups (p = 0.952). At the conclusion of the study, the steers offered WPCS, WPCSW, and SCS diets showed no difference for BW, of which was higher than that of beef cattle fed with CS diet (p<0.05). The DMI of beef cattle in 0 to 30 d and 61 to 90 d was different among the four treatments, as exhibited that was higher for steers offered the WPCS diet (p<0.05), whereas the difference was not significant from the whole period. The total ADG of beef cattle in WPCS group was significantly higher than that in WPCSW and SCS groups, while that in WPCSW and SCS groups was higher than that in CS group (p<0.001). There was no significant difference in F/G among WPCS, WPCSW, and SCS groups, but was lower in WPCS group than that in CS group (p = 0.039). Based on the above data, we evaluated the economic profit of the four diets. The results showed that the gross profit of WPCS diet was the highest, almost the same with SCS and WPCSW diet, of which were $1.81, 1.72, and 1.69 (dollar/head/d), respectively. However, the gross profit of CS diet was relatively low that is $1.44.

### Serum parameters

The effects of various corn silage-based diets on serum parameters of finishing beef cattle are reported in Table 5. The concentrations of ALB were higher for steers fed WPCS, WPCSW, and CS diets than the SCS diet (p = 0.024). However, no difference was observed in TP concentration among the steers fed four diets. The concentration of NEFA was higher for steers fed with CS and SCS diets than that in WPCS and WPCSW group steers (p<0.001). The steers in CS and SCS diet treatments showed higher LDH concentration compared with steers in WPCS diet (p<0.05). No significant difference was observed for the GLU, UN, and CREA concentrations.

### Meat nutrients

The general nutrient contents and amino acids composition of LD of steers are presented in Table 6 and 7. As is shown, the CP, EE, and organic compound contents were highest numerically for steers offered WPCS diet and lowest in steers offered CS diet, whereas the differences were not significant. Similarly, the corn silage-type did not affect the amino acids composition of LD.

## DISCUSSION

### Chemical composition of experimental silages and crop straws

The WPCS is an appropriate forage source for growing beef cattle, as it has a satisfactory chemical composition, with low fiber fraction content, higher starch concentration and good fermentation profile [9]. We determined the nutrient content of alternative forage sources to corn silage, including sweet corn stalklage, corn stalklage, wheat straw and peanut vine. The sweet corn stalklage showed relatively low nutrient components, such as concentrations of DM and starch, whereas the content of CP in sweet corn stalklage was highest among all forages used in current study. Corn stalk, a low-quality forage with limited feeding value, is the most abundant biomass worldwide and also is the major forage used in beef cattle diets [19]. Ensiling could improve its nutritional value. The fiber fraction in wheat straw was high, hence its inclusion in the diet was expected to reduce intake and performance, but it is particularly valuable when high quality feeds are insufficient or expensive. Peanut vine, a legume plant resource rich in CP content, is the main dry forage used in the present experiment. The selected forages are all very low in readily fermentable carbohydrates and degradable protein. However, some studies have confirmed that there is a synergistic effect when mixtures of whole crop cereal silages and leguminous plants are offered together, as compared to the silages offered alone [20].

### Ruminal degradability of experimental diets

Rumen degradability of nutrients is a major factor affecting diet intake, and thus animal performance. Due to economic and environmental concerns, the utilization of by-products of agricultural production such as crop straw in the diet is expected to increase and to become more efficient [21]. However, the nutritive values of these by-products are relatively low compared with that of WPCS. In our research, the ED of DM and CP were highest for the WPCS diet (40.82% and 46.16%, respectively), whereas the SCS diet showed higher ED of NDF (34.68%), and the WPCSW and SCS diets showed the same ED of ADF as the WPCS diet. These results explain the absence of differences in F/G across dietary treatments of WPCS, WPCSW, and SCS. Low-quality forage usually have low digestibility due to the decreased in ruminal degradability of plant cell walls, in which contain high concentrations of cell-wall polysaccharides and lignin [22]. The undegradable fraction for CS based diet was highest of all our roughages, this could be explained by the increase of physically effective fiber content, which reduced total-tract digestibility and thus increase F/G [23].

### Feed intake and growth performance

Nutrient intake and digestibility are the most important factors determining animal performance [24]. In the present study, the feeding trial continued for 90 days, in order to ensure the accuracy of the data, the diet compositions of steers were not adjusted throughout the trial. The beef cattle gradually matured, and growth rate dropped. This is a common growth pattern of beef cattle in feedlot. In addition, the trial was carried out in china from May to August, where the temperature gradually increases, which might result in the decline of steers’ intake due to heat stress. Therefore, the data of intake and growth were analyzed periodically by month to make the difference clear. The DMI of beef cattle in 61 to 90 d was higher for steers offered the WPCS diet, but not over the whole feeding trial, which might be attributed to the similar chemical composition of the four experimental diets. The ADG in WPCS group was significantly higher than that in WPCSW and SCS groups but there was no significant difference between WPCSW and SCS groups. In a similar study, He et al [3] stated that substituting corn stalklage with corn silage in a finishing ration did not affect the DMI and BW gain of cattle. These divergence results could be related to the quality of silages, as well as their inclusion levels in the diets. Forage quality and amounts are important for ruminal fermentation and cattle performance [25]. From the results of economic evaluation, although the gross profit of SCS and WPCSW diet were not higher than that of WPCS diet, they are almost the same in numerical value. The sweet stalklage and wheat straw could be used as a cheaper alternative in feedlot cattle diet, when the corn silage is in short supply, and without sharp reducing economic benefits.

### Serum biochemical indexes

To assess the health status and metabolism of beef steers, the relative blood biochemical parameters were measured. The concentration of NEFA was higher for steers fed with CS and SCS diets than for steers offered WPCS and WPCSW diets. The NEFA in blood is mainly an intermediate product of fat metabolism, of which is an important metabolic substrate for energy metabolism of body cells, providing energy for organ metabolism. Rosero [26] showed that NEFA is negatively correlated with digestible energy intake, but is affected by time of day, time after feeding, and very markedly by the amount of grain reaching the small intestine. In the current study, the four diet were formulated with similar forage to concentrate ratio, but the EE content was higher for CS and SCS diet (Table 2), which might normally increase NEFA concentration in serum that was conducive to promote energy utilization. The steers in CS and SCS diet treatments had a higher LDH concentration compared with steers fed the WPCS diet. The LDH is one of the most important oxidoreductases in glycolysis. It can catalyze reversibly the oxidation of lactic acid to pyruvate using the NAD^+^ coenzyme, which is the final product of anaerobic glycolysis. The increase of LDH activity indicated that steers fed CS and SCS diet relied more on glycolysis to provide energy to achieved the balance of energy metabolism. In addition, no significant differences were observed for TP, BUN, ALT, and CREA concentrations, suggesting that the types of diets in this trial had only low and transient effect on blood biochemical parameters.

### Meat nutrients

Generally, the moisture, protein, fat, mineral and vitamin are the most commonly measured nutritional components of beef muscle, and the contents of moisture, protein and fat in muscle are closely correlated and determine the sensory quality of beef [27]. In present study, the corn silage-type did not significantly affect the nutritional contents and amino acids composition in the LD. This is common, He et al [28] stated that cattle could be fed diets containing corn silage, corn stalk silage, or a mixture of corn stalk silage and corn grain during the finishing period with little difference in beef quality. The study of Walsh et al [29] showed that no major differences occurred in beef quality of cattle consuming maize silage, whole-crop cereals or concentrates. In general, compared to forages, concentrate feeding has the stronger impact on meat quality characteristics [30]. Our result stated that the stalk silage or wheat straw were feasible as an alternative forage used in beef cattle raising.

In conclusion, the corn silage-based diet exhibited the highest feeding value. The sweet stalklage and wheat straw as an alternative to corn silage offered to beef cattle had limited influence on feeding value and meat nutrients. However, the value of CS was relatively poor. To sum up, when the high quality forage resources, such as corn silage, are in short supply or the growth rate of beef cattle decreases in the later finishing period, the sweet stalklage and wheat straw could be used as a cheaper alternative in feedlot cattle diet without sharp reducing economic benefits.

## Figures and Tables

**Table 1:** Chemical composition of experimental silages and crop straws

Item	Corn silage			Dry crop straw	
	
	Whole plant corn silage	Sweet corn stalklage	Corn stalklage	Peanut vine	Wheat straw
Routine nutritive values (% of dry matter unless otherwise stated)					
Dry matter (% of fresh matter)	29.42	25.21	34.47	90.98	91.56
Crude protein	10.32	11.41	9.68	12.04	6.96
Ether extract	2.06	1.78	1.13	2.31	1.76
Neutral detergent fiber	48.36	50.57	62.72	46.92	75.66
Acid detergent fiber	30.38	36.35	39.36	35.95	52.17
Starch	28.56	19.37	13.48	-	-
Ash	4.33	4.26	6.18	9.98	8.26
Fermentation profile (% of dry matter unless otherwise stated)					
pH	3.82	4.14	4.53	-	-
Lactate	7.16	4.67	3.84		
Acetate	1.14	0.74	0.56	-	-
Propionate	0.11	0.08	0.07	-	-
Ammoniacal N/total N (%)	7.33	3.19	3.45	-	-

**Table 2:** Ingredients and chemical compositions of experimental diets

Items	Treatment			

	WPCS	WPCSW	CS	SCS
Ingredient (% of dry matter)				
Whole plant corn silage	24.24	14.65	0.00	0.00
Corn stalklage	0.00	0.00	23.06	0.00
Sweet corn stalklage	0.00	0.00	0.00	23.38
Peanut vine	17.98	18.11	17.78	18.63
Wheat straw	0.00	9.06	0.00	0.00
Dried distiller’s grains with solubles	12.18	11.65	12.04	10.40
Corn, ground	14.05	14.74	15.90	15.88
Tofukasu	0.56	0.57	0.56	0.57
Concentrate feed	30.99	31.22	30.66	31.14
Forage to concentrate ratio	42:58	42:58	41:59	42:58
Nutrient composition (% of dry matter unless otherwise stated)				
Dry matter (% of fresh matter)	51.91	51.51	52.48	50.63
NEmf^1)^ (Mcal/kg)	1.44	1.39	1.37	1.41
Crude protein	14.29	14.17	14.68	14.59
Ether extract	3.40	3.38	3.80	3.93
Neutral detergent fiber	34.70	37.79	36.52	33.66
Acid detergent fiber	24.34	25.72	24.78	22.66

WPCS, whole plant corn silage-based diet; WPCSW, mixed forages-based diet was formulated by substituting a portion of whole plant corn silage with wheat straw; CS, corn stalklage-based diet; SCS, sweet corn stalklage-based diet.

1) NEmf was calculated value, while the others were measured values.

**Table 3:** Ruminal degradability parameters of diets comprised of various corn silages

Items	Treatment				p-value

	WPCS	WPCSW	CS	SCS	
DM					
Soluble fraction (% of DM)	20.3±3.4^a^	12.7±3.0^b^	10.4±2.8^b^	19.5±2.8^a^	0.010
Slowly degradable fraction (% of DM)	68.1±5.2^b^	74.3±9.9^a^	75.9±10.7^ab^	69.9±5.0^b^	0.015
Undegradable fraction (% of DM)	11.6±1.4^b^	13.0±1.6^a^	13.7±1.6^a^	10.6±1.1^b^	0.017
Degradation rate (%/h)	2.3±0.3	1.6±0.7	2.1±0.8	2.5±0.7	0.288
Effective degradability (% of DM)	40.8±6.3^a^	32.2±4.1^c^	30.8±3.3^d^	34.5±5.0^b^	<0.001
CP					
Soluble fraction (% of CP)	20.9±3.0^a^	15.1±2.3^b^	14.3±1.3^b^	20.4±2.4^a^	0.029
Slowly degradable fraction (% of CP)	74.9±5.7	77.2±4.3	72.9±5.7	74.8±3.2	0.345
Undegradable fraction (% of CP)	4.2±0.2^c^	7.7±0.4^b^	12.8±0.9^a^	4.85±0.3^c^	0.032
Degradation rate (%/h)	2.3±0.4	2.5±0.7	2.5±0.7	2.4±0.2	0.837
Effective degradability (% of CP)	46.2±2.2^a^	39.4±5.7^c^	37.4±2.8^d^	43.7±3.0^b^	<0.001
NDF					
Soluble fraction (% of NDF)	11.4±1.6^a^	7.9±1.2^b^	7.1±1.8^b^	9.7±1.3^ab^	0.044
Slowly degradable fraction (% of NDF)	65.6±3.4^a^	71.2±2.1^a^	60.9±2.6^b^	69.5±4.5^a^	0.030
Undegradable fraction (% of NDF)	23.0±2.7^b^	20.9±2.6^b^	32.0±4.3^a^	20.8±2.1^b^	0.035
Degradation rate (%/h)	1.9±0.7	1.8±0.7	2.0±0.8	2.1±0.7	0.400
Effective degradability (% of NDF)	33.2±3.3^b^	31.0±2.6^c^	29.1±2.2^d^	34.7±2.3^a^	<0.001
ADF					
Soluble fraction (% of ADF)	12.8±2.5^a^	6.1±1.3^b^	6.1±1.27^b^	7.4±2.1^b^	0.027
Slowly degradable fraction (% of ADF)	59.4±5.4^b^	68.0±4.3^a^	60.0±4.03^b^	65.1±4.1^b^	0.042
Undegradable fraction (% of ADF)	27.8±3.1^b^	25.9±2.8^b^	33.9±3.6^a^	27.5±2.4^b^	0.023
Degradation rate (%/h)	1.9±0.711	1.6±0.7	2.1±0.5	2.6±0.6	0.276
Effective degradability (% of ADF)	33.2±1.9	27.1±3.0	29.8±1.3	29.8±2.1	0.052

WPCS, whole plant corn silage-based diet; WPCSW, mixed forages-based diet was formulated by substituting a portion of whole plant corn silage with wheat straw; SCS, sweet corn stalklage-based diet; CS, corn stalklage-based diet; DM, dry matter; CP, crude protein; NDF, neutral detergent fiber; ADF, acid detergent fiber.

a–d Means within a row with different superscripts differ (p<0.05).

**Table 4:** Feed intakes and growth performance of beef cattle offered alternative corn silage-based diets

Items	Period	Treatment				p-value

		WPCS	WPCSW	CS	SCS	
Body weight (kg)	0 d	427.64±25.34	429.03±25.98	425.86±28.05	428.15±26.58	0.952
	30 d	469.41±23.33	464.19±25.68	456.69±29.41	461.99±26.37	0.148
	60 d	496.12±22.36^a^	489.49±26.33^ab^	479.78±31.77^b^	486.57±26.86^ab^	0.040
	90 d	509.41±24.45^a^	501.75±26.73^ab^	490.64±31.49^b^	498.98±27.72^ab^	0.016
Dry matter intake (kg/d)	0 to 30 d	9.73±0.31^ab^	10.07±0.24^a^	9.50±0.32^b^	9.54±0.21^b^	<0.001
	31 to 60 d	8.96±0.34	8.70±0.58	8.53±0.31	8.69±0.47	0.427
	61 to 90 d	7.76±0.12^a^	7.31±0.22^b^	7.26±0.13^b^	7.26±0.10^b^	<0.001
	0 to 90 d	8.94±0.88	8.89±1.22	8.58±0.98	8.65±0.10	0.596
Average daily gain (kg/d)	0 to 30 d	1.27±0.33^a^	1.07±0.17^b^	0.93±0.21^c^	1.03±0.18^bc^	<0.001
	31 to 60 d	0.83±0.21	0.79±0.12	0.72±0.34	0.77±0.10	0.092
	61 to 90 d	0.58±0.65	0.53±0.80	0.47±0.31	0.54±0.66	0.885
	0 to 90 d	0.93±0.26^a^	0.83±0.22^b^	0.74±0.16^c^	0.80±0.19^b^	<0.001
Feed conversion ratio (F/G)	0 to 30 d	7.69±0.24^c^	9.44±0.22^b^	10.16±0.34^a^	9.30±0.20^b^	<0.001
	31 to 60 d	10.73±0.40^b^	11.01±0.74^b^	11.83±0.43^a^	11.31±0.61^ab^	0.019
	61 to 90 d	13.42±0.21^c^	13.71±0.42^b^	15.38±0.27^a^	13.46±0.19^bc^	<0.001
	0 to 90 d	10.20±2.47^b^	11.11±1.87^ab^	12.13±2.24^a^	11.06±1.87^ab^	0.039
Gross profit (dollar)1)	0 to 90 d	1.81	1.72	1.44	1.69	-

WPCS, whole plant corn silage-based diet; WPCSW, mixed forages-based diet was formulated by substituting a portion of whole plant corn silage with wheat straw; SCS, sweet corn stalklage-based diet; CS, corn stalklage-based diet.

p-value for the main effect of treatment (type of diet).

1) The gross price of beef cattle was 3.82 dollars, Gross profit = income from weight gain – expense of feed.

a–c Means within a row with different superscripts differ (p<0.05).

**Table 5:** Serum parameters of beef cattle offered alternative corn silage-based diets

Items	Treatment				p-value

	WPCS	WPCSW	CS	SCS	
Glucose (mmol/L)	5.36±0.31	5.28±0.19	5.17±0.52	5.08±0.96	0.284
Total protein (g/L)	66.56±2.41	66.36±4.79	63.59±4.54	66.40±3.31	0.321
Albumin (g/L)	37.47±2.20^a^	35.70±2.48^ab^	35.44±3.21^ab^	33.83±0.90^b^	0.024
Globulin (g/L)	29.10±3.78	30.65±3.88	28.16±3.59	32.58±3.76	0.087
Albumin/globulin	1.31±0.22^a^	1.18±0.18^ab^	1.28±0.22^a^	1.05±0.16^b^	0.038
Urea nitrogen (mmol/L)	7.65±0.78	7.51±0.98	7.70±1.52	7.57±0.67	0.982
Creatinine (μmol/L)	109.12±16.80	109.88±12.42	102.62±11.01	107.62±11.53	0.648
Nonesterified fatty acid (mmol/L)	0.15±0.02^b^	0.14±0.012^b^	0.20±0.01^a^	0.18±0.03^a^	<0.001
Lactate dehydrogenase (U/L)	5,507.75±266.28^c^	5,698.11±405.38^bc^	6,035.18±145.95^a^	5,920.22±382.33^ab^	0.006

WPCS, whole plant corn silage-based diet; WPCSW, mixed forages-based diet was formulated by substituting a portion of whole plant corn silage with wheat straw; SCS, sweet corn stalklage-based diet; CS, corn stalklage-based diet.

a–c Means within a row with different superscripts differ (p<0.05).

**Table 6:** Nutrient content of Longissimus dorsi of cattle offered alternative corn silage-based diets

Items	Treatment				p-value

	WPCS	WPCSW	CS	SCS	
Moisture (% of fresh matter)	71.40±3.78	74.37±3.77	76.52±3.61	74.53±3.35	0.210
Crude protein (% of dry matter)	21.39±2.47	19.75±2.65	18.86±2.74	19.26±2.32	0.442
Ether extract (% of dry matter)	6.24±3.49	4.92±1.41	3.86±1.12	5.49±1.57	0.368
Organic compound (% of dry matter)	27.57±3.74	24.70±3.63	22.56±3.47	24.60±3.26	0.207
Ca (% of dry matter)	0.04±0.01	0.03±0.01	0.03±0.01	0.03±0.01	0.900
P (% of dry matter)	0.03±0.01	0.04±0.01	0.04±0.01	0.04±0.01	0.768

WPCS, whole plant corn silage-based diet; WPCSW, mixed forages-based diet was formulated by substituting a portion of whole plant corn silage with wheat straw; SCS, sweet corn stalklage-based diet; CS, corn stalklage-based diet.

**Table 7:** Amino acid profile of Longissimus dorsi of beef cattle offered alternative corn silage-based diets

Items	Treatment				p-value

	WPCS	WPCSW	CS	SCS	
Asparagine	3.70±1.56	4.22±2.70	4.83±3.83	3.11±1.05	0.739
Glutamine	7.86±1.87	7.32±2.07	6.48±5.02	6.23±2.05	0.822
Serine	1.92±0.45	1.84±0.52	1.58±1.16	1.57±0.59	0.829
Glycine	3.22±0.91	3.93±0.97	2.69±1.54	2.96±1.20	0.794
Histidine	2.58±0.72	2.68±0.95	2.14±1.52	2.21±0.95	0.820
Arginine	4.18±0.96	4.08±1.08	3.40±2.26	3.42±1.05	0.743
Threonine	2.40±0.53	2.31±0.58	1.93±1.38	2.13±0.91	0.857
Alanine	3.95±0.99	4.06±1.08	3.16±1.73	3.36±0.94	0.596
proline	4.04±1.30	4.19±1.23	3.22±1.40	4.00±1.93	0.736
Tyrosine	4.94±2.05	3.85±0.80	3.05±1.35	4.66±1.34	0.205
Valine	1.14±0.31	1.08±0.20	0.83±0.49	1.04±0.42	0.713
Methionine	3.66±0.96	3.67±0.98	2.98±1.77	3.24±0.91	0.766
Cysteine	1.02±0.36	0.95±0.28	0.80±0.48	0.86±0.22	0.748
Isoleucine	2.69±0.71	2.75±0.68	2.24±1.29	2.61±0.84	0.817
Leucine	5.59±1.79	5.59±1.56	4.54±2.38	5.21±1.67	0.794
Phenylalanine	2.17±0.62	2.18±0.56	1.74±0.92	2.18±0.83	0.729
Lysine	3.79±1.32	3.44±0.92	2.73±1.84	3.65±1.14	0.621
Essential amino acid	21.44±5.89	21.03±5.32	17.01±10.01	20.07±6.76	0.766
Total amino acid	58.85±14.09	57.60±15.58	48.36±29.75	52.45±15.45	0.825

WPCS, whole plant corn silage-based diet; WPCSW, mixed forages-based diet was formulated by substituting a portion of whole plant corn silage with wheat straw; CS, corn stalklage-based diet; SCS, sweet corn stalklage-based diet.
